# Supplementary material for: Prognostic Value of Copy Number Alteration Burden in Early-Stage Breast Cancer and the Construction of an 11-Gene Copy Number Alteration Model
Source: Cancers (Basel). 2022 Aug 27;14(17):4145. doi: 10.3390/cancers14174145 (PMC9454926; doi:10.3390/cancers14174145)
Supplement: Supplementary file 1 [file cancers-14-04145-s001.zip › Supplementary Figures.pdf]

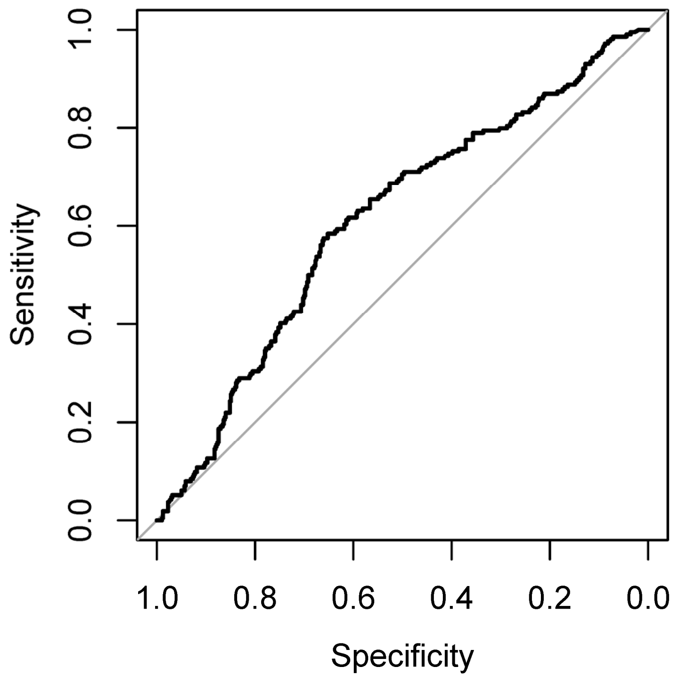

Supplementary Figure S1 Receiver operating characteristic curve of the 5-year relapse-free survival of patients in the METABRIC cohort

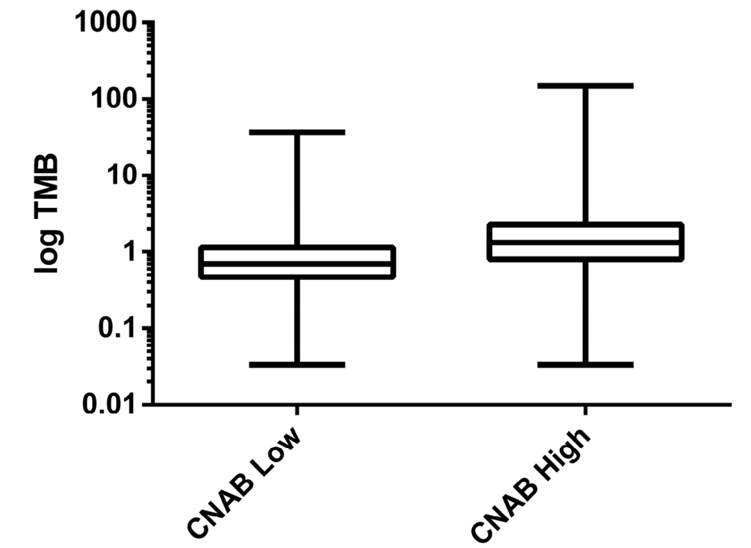

Supplementary Figure S2 Tumor mutational burden of patients in different copy number alteration burden groups

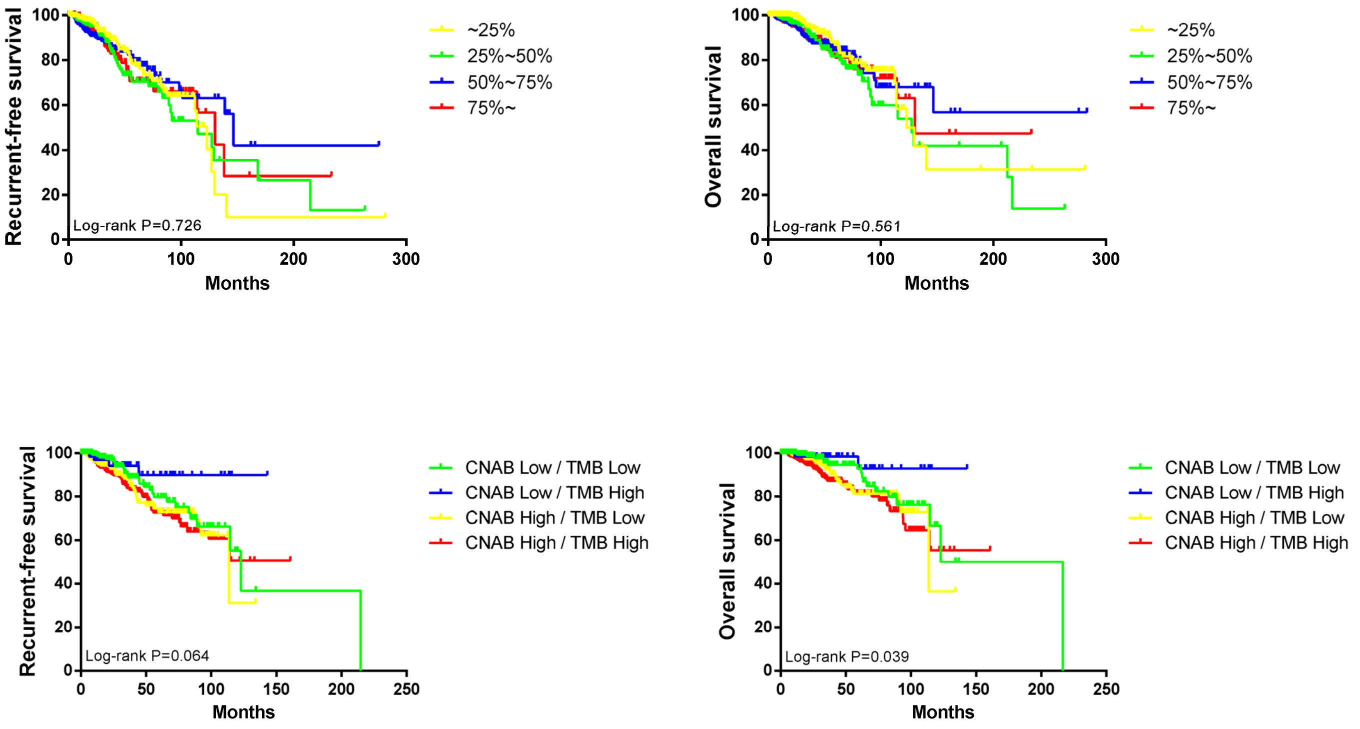

Supplementary Figure S3 Survival analysis for different tumor mutational burden groups and combined survival analysis of tumor mutational burden and copy number alteration burden of patients in TCGA cohort.

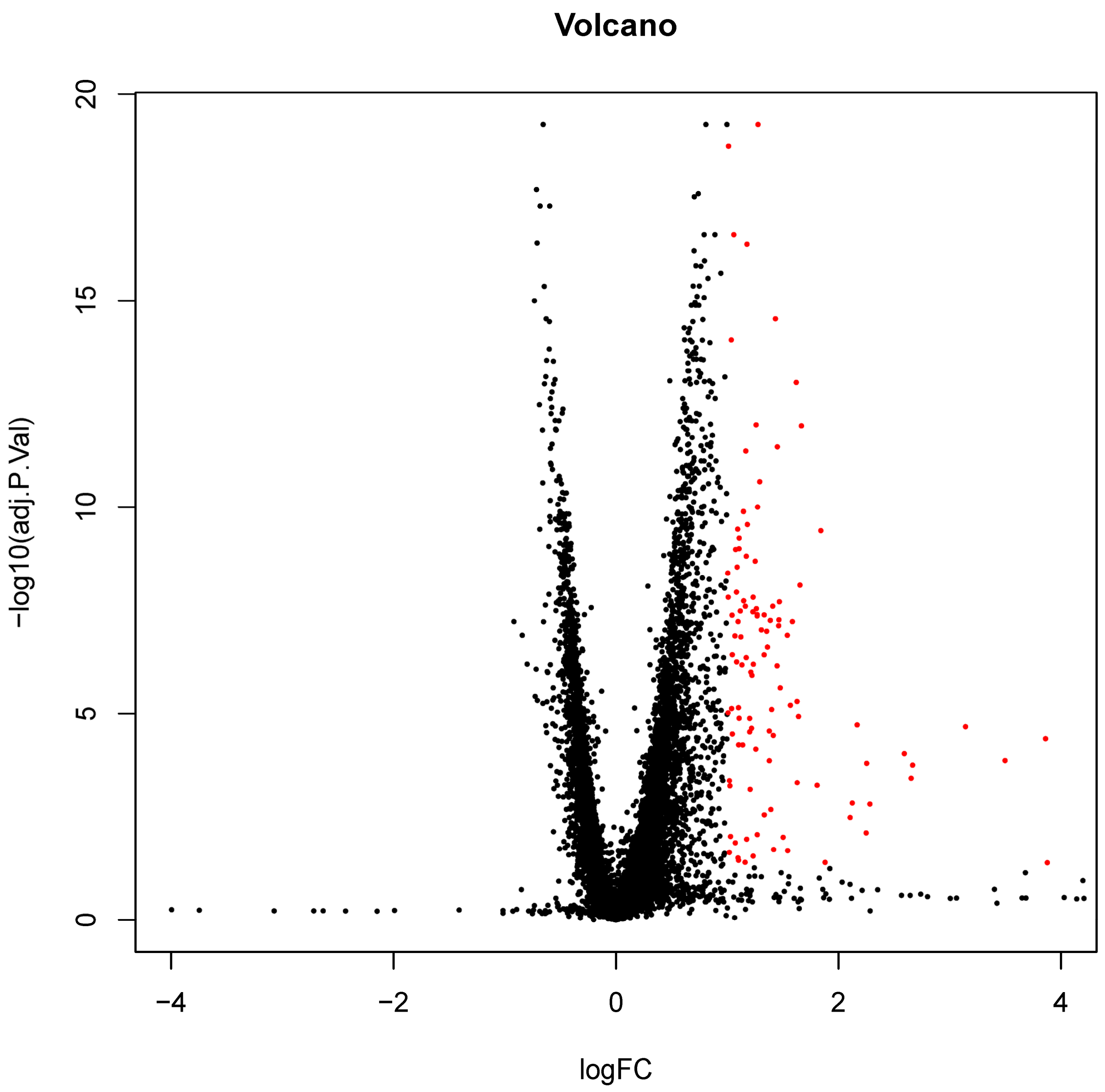

Supplementary Figure S4 Volcano plot of the expression profile difference between the CNAB11 high group and the CNAB11 low group.
