# Supplementary material for: Prognostic Value of Copy Number Alteration Burden in Early-Stage Breast Cancer and the Construction of an 11-Gene Copy Number Alteration Model
Source: Cancers (Basel). 2022 Aug 27;14(17):4145. doi: 10.3390/cancers14174145 (PMC9454926; doi:10.3390/cancers14174145)
Supplement: Supplementary file 1 [file cancers-14-04145-s001.zip › Supplementary Method.pdf]

Supplementary Method

DNA extraction

All tissue sections were reviewed by two breast pathologists. Tumor areas with an estimated minimum tumor cell percentage of 30% were selected. Ten consecutive FFPE tissue sections (5-μm-thick) were deparaffinized and hematoxylin stained, followed by microdissection. Selected tumor areas and normal tissue areas were microdissected respectively by using a sterile scalpel prior to adding 5% Chelex 100 Resin (Bio-Rad, Hercules, CA, USA) Cell lysis solution (Promega, Madison, WI, USA). DNA of the two samples above was extracted by proteinase K (Roche, Mannheim, Germany) digestion incubated overnight at 56 °C. Proteinase K was inactivated at 95 °C for 10 min. Finally, the samples were centrifuged for 5 min at 20 000 g to remove the remaining cell debris and Chelex resins. The DNA was collected into new tubes and then stored at -80 °C for further use. DNA concentrations were measured by a Qubit 2.0 fluorometer (Thermo Fisher Scientific, Waltham, MA, USA).

Targeted next-generation sequencing

The Ion AmpliSeq Designer tool was used to design amplicons for the multiplex PCR assay aiming for 150-bp amplicons, therehy allowing efficient amplification for fragmented DNA isolated from FFPE tissues. Full sequence coverage of large exons required amplification and sequencing of overlapping amplicons. Therefore, the multiplexed PCR was divided into two reactions, using 10 ng DNA for each reaction. The Ion AmpliSeq Library Kit Plus (Thermo Fisher Scientific) protocol was used to process the samples analyzed by the Ion AmpliSeq custom panel, following the manufacturer's instructions. Each sample was barcoded by IonXpress barcode adapters, allowing multiplexed sequencing. A total of 18 PCR cycles were performed. All samples were multiplexed on an Ion 540 Chip and sequenced on an Ion S5XL Semiconductor Sequencer (Thermo Fisher Scientific, Waltham, MA, USA).
