# Supplementary material for: Prognostic Value of Copy Number Alteration Burden in Early-Stage Breast Cancer and the Construction of an 11-Gene Copy Number Alteration Model
Source: Cancers (Basel). 2022 Aug 27;14(17):4145. doi: 10.3390/cancers14174145 (PMC9454926; doi:10.3390/cancers14174145)
Supplement: Supplementary file 1 [file cancers-14-04145-s001.zip › Supplementary Tables S1-S7.pdf]

Supplementary Table S1 Clinical characteristics differences between the high and low CNAB groups of the METABRIC cohort

	Training Cohort					Test Cohort				
	CNAB Low		CNAB High		P	CNAB Low		CNAB High		P
	Cases	%	Cases	%		Cases	%	Cases	%	
Overall	416		298			418		295		
Age					0.276					0.753
<50	173	41.59	137	45.97		195	46.65	142	48.14	
≥50	243	58.41	161	54.03		223	53.35	153	51.86	
Subtype					<0.001					<0.001
HR+HER2-	339	81.49	176	59.06		341	81.58	169	57.29	
HER2+	33	7.93	52	17.45		34	8.13	58	19.66	
TNBC	44	10.58	70	23.49		43	10.29	68	23.05	
Grade					<0.001					<0.001
I	51	12.26	8	2.68		53	12.68	4	1.36	
II	208	50	74	24.83		195	46.65	72	24.41	
III	139	33.41	211	70.81		151	36.12	214	72.54	
Stage					0.018					<0.001
I	163	39.18	92	30.87		165	39.47	76	25.76	
II	219	52.64	174	58.39		233	55.74	190	64.41	
III	34	8.17	32	10.74		20	4.78	29	9.83	
Radiotherapy					0.088					0.007
No	145	34.86	85	28.52		167	39.95	88	29.83	
Yes	271	65.14	213	71.48		251	60.05	207	70.17	
Chemotherapy					<0.001					0.002
No	355	85.34	213	71.48		349	83.49	202	68.47	
Yes	61	14.66	85	28.52		69	16.51	93	31.53	
Hormone Therapy					0.154					0.186
No	150	36.06	124	41.61		154	36.84	124	42.03	
Yes	266	63.94	174	58.39		264	63.16	171	57.97	
Surgery					0.389					0.818

Lumpectomy	192	46.15	127	42.62	172	41.15	118	40
Mastectomy	224	53.85	171	57.38	246	58.85	177	60

Supplementary Table S2 Baseline differences between the high and low CNAB groups of TCGA cohort

	CNAB Low		CNAB High		P
	Cases	%	Cases	%	
Overall	251		586		
Age					0.369
<50	127	50.6	318	54.27	
≥50	124	49.4	268	45.73	
Subtype					<0.001
HR+HER2-	213	84.86	314	53.58	
HER2+	27	10.76	147	25.09	
TNBC	11	4.38	125	21.33	
Stage					0.006
I	61	24.3	86	14.68	
II	138	54.98	356	60.75	
III	52	20.72	144	24.57	
Radiotherapy					0.982
No	120	47.81	278	47.44	
Yes	131	52.19	308	52.56	
Drug therapy					0.158
No	226	90.04	546	93.17	
Yes	25	9.96	40	6.83	
Surgery					0.596
Lumpectomy	68	27.09	126	21.5	
Mastectomy	124	49.4	258	44.03	

Supplementary Table S3 Univariate and multivariate Cox regression model on METABRIC test cohort

	Recurrent-free survival				Overall survival			
	Univariate Cox regression		Multivariate Cox regression		Univariate Cox regression		Multivariate Cox regression	
	HR	*P*	HR	*P*	HR	*P*	HR	*P*
CNAB								
Low	1 (reference)		1 (reference)		1 (reference)		1 (reference)	
High	1.44 (1.19-1.74)	<0.001	1.33 (1.08-1.63)	0.008	1.46 (1.2-1.79)	<0.001	1.25 (1.01-1.56)	0.042
Age								
<60	1 (reference)		1 (reference)		1 (reference)		1 (reference)	
≥60	1.69 (1.39-2.06)	<0.001	1.62 (1.32-1.99)	<0.001	2.15 (1.74-2.66)	<0.001	2.09 (1.68-2.6)	<0.001
Subtype								
HER2+	1 (reference)		1 (reference)		1 (reference)		1 (reference)	
HR+HER2-	0.63 (0.48-0.83)	<0.001	0.64 (0.48-0.86)	0.003	0.56 (0.42-0.73)	<0.001	0.56 (0.42-0.75)	<0.001
TNBC	0.57 (0.4-0.82)	0.002	0.57 (0.39-0.81)	0.002	0.62 (0.43-0.88)	0.009	0.6 (0.42-0.87)	0.007
Grade								
I	1 (reference)		1 (reference)		1 (reference)		1 (reference)	
II	1.4 (0.92-2.12)	0.115	1.05 (0.69-1.6)	0.819	1.46 (0.93-2.3)	0.096	1.01 (0.64-1.6)	0.958
III	1.63 (1.08-2.44)	0.019	1.02 (0.66-1.57)	0.941	1.82 (1.17-2.83)	0.008	0.99 (0.62-1.59)	0.973
Stage								
I	1 (reference)		1 (reference)		1 (reference)		1 (reference)	
II	1.59 (1.28-1.97)	<0.001	1.45 (1.16-1.82)	0.001	1.81 (1.44-2.28)	<0.001	1.62 (1.27-2.06)	<0.001
III	3.04 (2.1-4.38)	<0.001	2.82 (1.94-4.1)	<0.001	3.62 (2.48-5.28)	<0.001	3.2 (2.17-4.71)	<0.001
Radiotherapy								
No	1 (reference)				1 (reference)			
Yes	0.89 (0.73-1.08)	0.239			0.93 (0.76-1.13)	0.455		
Chemotherapy								
No	1 (reference)				1 (reference)			
Yes	1.11 (0.88-1.41)	0.382			1.17 (0.91-1.5)	0.211		
Hormone Therapy								
No	1 (reference)				1 (reference)			

Yes	1.06 (0.87-1.28)	0.575			1.21 (0.98-1.48)	0.072		
Surgery								
Lumpectomy	1 (reference)		1 (reference)		1 (reference)		1 (reference)	
Mastectomy	1.57 (1.29-1.92)	<0.001	1.45 (1.18-1.79)	<0.001	1.63 (1.32-2.01)	<0.001	1.45 (1.17-1.81)	<0.001

Supplementary Table S4 Univariate and multivariate Cox regression model on TCGA cohort

	Recurrent-free survival				Overall survival			
	Univariate Cox regression		Multivariate Cox regression		Univariate Cox regression		Multivariate Cox regression	
	HR	*P*	HR	*P*	HR	*P*	HR	*P*
CNAB								
Low	1 (reference)		1 (reference)		1 (reference)		1 (reference)	
High	1.62 (1.08-2.46)	0.021	1.68 (1.11-2.54)	0.015	1.94 (1.15-3.28)	0.013	2.28 (1.19-4.4)	0.014
Age								
<60	1 (reference)		1 (reference)		1 (reference)		1 (reference)	
≥60	1.95 (1.36-2.78)	<0.001	2.07 (1.44-2.97)	<0.001	2.41 (1.55-3.75)	<0.001	2.39 (1.35-4.25)	0.003
Subtype								
HER2+	1 (reference)				1 (reference)			
HR+HER2-	0.75 (0.47-1.17)	0.205			0.6 (0.35-1.03)	0.065		
TNBC	1.25 (0.74-2.11)	0.408			1.17 (0.63-2.18)	0.61		
Stage								
I	1 (reference)		1 (reference)		1 (reference)		1 (reference)	
II	1.42 (0.83-2.42)	0.206	1.39 (0.81-2.39)	0.233	1.39 (0.71-2.72)	0.331	1.84 (0.63-5.42)	0.268
III	3.42 (1.95-6.01)	<0.001	4.84 (2.71-8.62)	<0.001	3.61 (1.81-7.17)	<0.001	10.65 (3.41-33.21)	<0.001
Radiotherapy								
No	1 (reference)		1 (reference)		1 (reference)		1 (reference)	
Yes	0.52 (0.37-0.75)	<0.001	0.41 (0.28-0.59)	<0.001	0.31 (0.2-0.49)	<0.001	0.25 (0.13-0.48)	<0.001
Drug therapy								
No	1 (reference)				1 (reference)			
Yes	1.14 (0.6-2.18)	0.696			1.05 (0.46-2.41)	0.914		
Surgery								
Lumpectomy	1 (reference)				1 (reference)		1 (reference)	
Mastectomy	1.31 (0.82-2.1)	0.261			1.92 (1-3.67)	0.048	1.01 (0.5-2.07)	0.973

Supplementary Table S5 Sensitivity Analysis

	Recurrent-free survival		Overall survival	
	HR	*P*	HR	*P*
METABRIC test cohort				
Cut-off=1	1.26 (1.00-1.59)	0.052	1.40 (1.09-1.80)	0.009
Cut-off=1.4	1.26 (1.03-1.54)	0.028	1.27 (1.03-1.57)	0.028
Cut-off=1.8	1.34 (1.11-1.62)	0.002	1.40 (1.15-1.71)	<0.001
Cut-off=2.2	1.44 (1.19-1.74)	<0.001	1.46 (1.20-1.79)	<0.001
Cut-off=2.6	1.40 (1.15-1.70)	<0.001	1.43 (1.16-1.76)	<0.001
Cut-off=3	1.21 (0.97-1.5)	0.090	1.25 (1.00-1.57)	0.053
TCGA				
Cut-off=1	1.34 (0.70-2.55)	0.379	1.51 (0.66-3.46)	0.334
Cut-off=1.4	1.38 (0.84-2.28)	0.203	2.32 (1.12-4.80)	0.024
Cut-off=1.8	1.46 (0.94-2.26)	0.094	1.75 (1.00-3.08)	0.051
Cut-off=2.2	1.62 (1.08-2.46)	0.021	1.94 (1.15-3.28)	0.013
Cut-off=2.6	1.50 (1.02-2.20)	0.041	2.06 (1.25-3.42)	0.005
Cut-off=3	1.44 (0.99-2.09)	0.054	2.17 (1.33-3.54)	0.002

Supplementary Table S6 Subgroup survival analysis on RFS

	Cases	Events	Univariate Cox regression		Multivariate Cox regression	
			HR	*P*	HR	*P*
**METABRIC**						
Overall	713	431	1.44 (1.19-1.74)	<0.001	1.33 (1.08-1.63)	0.008
Age						
<60	337	157	2.04 (1.49-2.79)	<0.001	1.83 (1.3-2.59)	<0.001
⩾60	376	274	1.12 (0.88-1.43)	0.355	1.11 (0.85-1.46)	0.439
Subtype						
HR+HER2-	510	309	1.44 (1.14-1.82)	0.002	1.29 (1-1.66)	0.047
HER2+	92	66	1.55 (0.92-2.59)	0.097	1.44 (0.82-2.52)	0.2
TNBC	111	56	1.22 (0.71-2.12)	0.473	1.08 (0.6-1.96)	0.796
Stage						
I	241	119	1.15 (0.78-1.69)	0.485	1.11 (0.72-1.71)	0.634
II	423	274	1.39 (1.1-1.76)	0.007	1.37 (1.05-1.78)	0.019
III	49	38	1.55 (0.79-3.04)	0.199	1.89 (0.89-4.04)	0.098
**TCGA**						
Overall	837	124	1.62 (1.08-2.46)	0.021	1.68 (1.11-2.54)	0.015
Age						
<60	445	55	1.48 (0.79-2.77)	0.215	1.57 (0.84-2.95)	0.156
⩾60	392	69	1.93 (1.1-3.37)	0.021	1.74 (0.99-3.06)	0.52
Stage						
I	147	17	1.51 (0.52-4.35)	0.448	1.6 (0.55-4.61)	0.386
II	494	62	1.37 (0.76-2.45)	0.293	1.44 (0.8-2.59)	0.218
III	196	45	2.12 (1.02-4.442)	0.044	1.86 (0.89-3.9)	0.098

Supplementary Table S7 Subgroup survival analysis on OS

	Cases	Events	Univariate Cox regression		Multivariate Cox regression	
			HR	*P*	HR	*P*
**METABRIC**						
Overall	713	392	1.46 (1.2-1.79)	<0.001	1.25 (1.01-1.56)	0.042
Age						
<60	337	128	2.22 (1.57-3.15)	<0.001	1.77 (1.21-2.58)	0.003
⩾60	376	264	1.14 (0.89-1.46)	0.286	1.08 (0.82-1.43)	0.577
Subtype						
HR+HER2-	510	274	1.4 (1.1-1.79)	0.007	1.21 (0.93-1.58)	0.164
HER2+	92	63	1.45 (0.85-2.45)	0.169	1.39 (0.78-2.47)	0.265
TNBC	111	55	1.33 (0.76-2.33)	0.316	1.16 (0.63-2.11)	0.638
Stage						
I	241	104	1.31 (0.87-1.97)	0.196	1.26 (0.79-1.98)	0.329
II	423	251	1.33 (1.04-1.7)	0.024	1.23 (0.94-1.61)	0.13
III	49	37	1.48 (0.75-2.91)	0.255	1.46 (0.7-3.07)	0.314
**TCGA**						
Overall	837	84	1.94 (1.15-3.28)	0.013	2.28 (1.19-4.4)	0.014
Age						
<60	445	34	1.78 (0.77-4.11)	0.175	1.88 (0.81-4.36)	0.144
⩾60	392	50	2.4 (1.2-4.79)	0.013	2.26 (1.11-4.62)	0.025
Stage						
I	147	11	1.03 (0.29-3.66)	0.965	1.06 (0.3-3.78)	0.92
II	494	41	2.08 (0.92-4.7)	0.078	2.24 (0.98-5.09)	0.055
III	196	32	2.38 (0.97-5.8)	0.058	2.07 (0.84-5.09)	0.114
